# Human resources for health and maternal mortality in Latin America and the Caribbean over the last three decades: a systemic-perspective reflections

**DOI:** 10.1186/s12939-024-02154-y

**Published:** 2024-04-01

**Authors:** Gustavo Nigenda, Edson Serván-Mori

**Affiliations:** 1 Faculty of Nursing and Obstetrics, National Autonomous University of Mexico, Mexico City, Mexico; 2 Center for Health Systems Research, National Institute of Public Health of Mexico, Universidad Av. 655, Cuernavaca, Morelos, Mexico

**Keywords:** Human resources in health, Planning, Maternal health, Systemic perspective, Latin America and the Caribbean

## Abstract

**Background:**

The role of human resources for health in the operation of health systems is crucial. However, training and incorporating them into institutions is a complex process due to the continuous misalignment between the supply and demand of health personnel. Taking the case of the Latin American and Caribbean region countries, this comment discusses the relationship between the availability of human resources for health and the maternal mortality ratio for the period 1990–2021. It proposes the need to resume planning exercises from a systemic perspective that involves all areas of government and the private sector linked to the training and employment of health workers.

**Main text:**

We used secondary data from a global source to show patterns in the relationship between these two aspects and identify gaps in the Latin American and Caribbean regions. The results show enormous heterogeneity in the response of regional health systems to the challenge of maternal mortality in the region. Although most countries articulated specific programs to achieve the reduction committed by all countries through the Millennium Development Goals, not all had the same capacity to reduce it, and practically none met the target. In addition, in the English Caribbean countries, we found significant increases in the number of health personnel that do not explain the increases in the maternal mortality rate during the period.

**Conclusions:**

The great lesson from the data shown is that some countries could articulate responses to the problem using available resources through effective strategies, considering the specific needs of their populations. Although variations in maternal mortality rate cannot be explained solely through the provision of health personnel, it is important to consider that it is critical to find new modalities on how human resources for health could integrate and create synergies with other resources to increase systems capacity to deliver care according to conditions in each country.

## Background

 Despite reaching levels of over 85% in the coverage of skilled birth attendance in Latin America and the Caribbean (LAC) between 1990 and 2015 [1], the trajectory followed by the maternal mortality ratio (MMR) in those years has yet to be satisfactory. The generalized inability of the region’s countries to achieve target 5 A of the Millennium Development Goals (MDGs) to reduce the MMR by 75% expresses the enormous difficulties that this represented [2].

Maternal mortality is a crucial tracer for assessing the state of social inequality and inequities and the performance of health systems [3]. Its close relationship with poverty and its multifactorial nature [4–8] make it a social phenomenon with determinants that transcend health systems [8], and its effective mitigation requires a more profound understanding from a systemic, not insular, perspective [9].

The role of human resources for health (HRH) in health systems is a recurrent topic of interest among planners and decision-makers at the global level [10]. However, there is no consensus on organizing the health workforce to optimize its response to population needs [11]. The recent COVID-19 pandemic highlighted the complex interdependence between the different social subsystems (health, labor, education, politics, and economics), their underlying resources and processes [12], and the need to consider the role and management of HRH [13]. However, beyond the lessons and sequelae that this pandemic will leave in health systems, the management of HRH, back to routine conditions, will continue to be complex it have to contend with the coexistence of the growing burden of both pre-transitional and post-transitional diseases [11].

Optimizing sexual and reproductive health outcomes is a current challenge for low- and middle-income countries (LMICs), with direct implications for the achievement not only of goal 3 (health and well-being) of the Sustainable Development Goals (SDGs) but also goals 4 (education and quality), 5 (gender equality) and 10 (reduction of inequalities) [14], mainly because their most negative manifestations are concentrated in socially disadvantaged population groups [5–8, 15–19]. In particular, addressing the fragility and unsustainability of the achievements in maternal mortality implies rethinking whether the current care and HRH management models are viable and relevant or whether it is necessary to reconsider their structure [20].

Based on an ecological and descriptive analysis, this comment discusses, from a systemic perspective [21], the evolution of the MMR (maternal deaths per 100,000 live births in women aged 15–49 years) for 1990–2021, its relationship with the provision of HRH among LAC countries, and the implications of this relationship for the health systems of LAC and other regions of the world. All this is a basis for proposing strategies that seek to close existing gaps identified by the MDGs in 2000, the SDGs in 2015, and COVID-19 initiated in 2020. To this end, we conducted a multi-country analysis for LAC using data for the period 1990–2021, recently updated by the Global Burden of Disease (GBD) study [22–24], which provides guidelines for the presentation of accurate and transparent health estimates (GATHER) [25] as well as historical data on the burden of disease in countries even at the subnational level [22]. Further methodological details and the analytical scope of these data can be found in other publications [22–24]. The analyzed data were downloaded from the websites https://vizhub.healthdata.org/sdg/#0 and http://ghdx.healthdata.org/record/ihme-data/gbd-2017-health-related-sdgs-1990-2030 during November 2023.

Specifically, we present estimates of MMR and HRH (physician, nurses, and midwives) availability for 1990, 2000 (signing of the MDGs), 2015 (end of the MDG era and start of the SDGs), 2019 (pre-COVID-19) and 2021 (two years from the beginning of COVID-19), and describe their temporal dynamics through percentage change and average annual growth rates (or speed of change), as well as changes in regional gaps. We also present the physician/nurse and midwife ratios of the metrics above. Finally, we relate changes in MMR and HRH staffing.

## Evolution of maternal mortality in LAC and HRH availability

The data analyzed showed a 30.9% reduction in MMR 1990–2019 (at a rate of 1.1% per year), a change that was reduced after the first two years of COVID-19, to 28.7%, from 132.9 in 1990 to 94.7 in 2021 (Table 1). In 1990, the countries with the highest levels of MMR were Haiti (467.9), Bolivia (345.6), Guatemala (260.2), Honduras (238.0), Peru (203.8), and El Salvador (187.8). In contrast, the countries with the lowest MMR in 1990 were Costa Rica (30.0), Antigua and Barbuda (30.6), Uruguay (31.3), Belize (31.9) and Jamaica (32.0). The countries with the most considerable reductions in MMR were El Salvador (74.9%), Brazil (51.9%), Honduras (51.4%), Peru (50.8%) and Bolivia (37.2%). These countries also recorded the highest reduction rates (4.2%, 2.3%, 2.2%, 2.2% and 1.4% per year). Fifteen of the 32 countries analyzed recorded increases of 3 to more than 150% in MMR.

**Table 1:** Level and change in maternal mortality ratio in LAC, 1990–2021

Country	Maternal mortality ratio				Relative change, %			Average annual growth rate (1990–2021), %
	1990	2015	2019	2021	1990–2015	1990–2019	1990–2021	
Haiti	467.9	494.1	452.2	434.1	5.6	-3.4	-7.2	-0.2
Bolivia	345.6	208.1	208.2	217.0	-39.8	-39.8	-37.2	-1.4
Guatemala	260.2	173.5	182.9	168.8	-33.3	-29.7	-35.1	-1.3
Honduras	238.0	114.3	109.5	115.6	-52.0	-54.0	-51.4	-2.2
Peru	203.8	83.5	112.1	100.3	-59.0	-45.0	-50.8	-2.2
El Salvador	187.8	52.3	45.0	47.1	-72.1	-76.1	-74.9	-4.2
Brazil	141.7	60.4	59.6	68.2	-57.4	-57.9	-51.9	-2.3
Ecuador	135.8	76.1	96.4	88.4	-44.0	-29.0	-34.9	-1.3
Paraguay	111.5	94.3	108.5	104.2	-15.4	-2.7	-6.5	-0.2
Suriname	97.7	139.6	143.0	141.2	42.8	46.3	44.4	1.2
Colombia	95.3	93.4	77.6	76.6	-2.0	-18.6	-19.6	-0.7
Dominican Republic	90.7	121.3	126.1	96.3	33.7	39.0	6.1	0.2
Guyana	82.2	160.4	156.2	161.7	95.2	90.1	96.8	2.1
Nicaragua	77.0	57.1	53.5	49.4	-25.8	-30.4	-35.8	-1.4
Mexico	74.6	43.7	47.8	53.0	-41.4	-35.9	-28.9	-1.1
Venezuela	72.3	81.6	114.2	129.7	12.8	58.0	79.4	1.8
Panama	72.1	72.1	68.3	71.7	0.1	-5.2	-0.5	0.0
Argentina	65.4	50.6	57.1	55.1	-22.6	-12.7	-15.8	-0.5
Trinidad and Tobago	59.0	87.0	91.1	95.5	47.4	54.4	61.8	1.5
Cuba	55.2	51.5	53.5	55.6	-6.6	-3.1	0.8	0.0
Bahamas	53.0	103.9	106.7	115.7	96.0	101.2	118.3	2.5
Saint Vincent and the Grenadines	48.2	90.3	92.9	102.4	87.2	92.6	112.3	2.4
Barbados	46.9	67.3	65.0	57.8	43.6	38.7	23.2	0.7
Saint Lucia	46.9	90.6	91.5	96.3	93.2	95.3	105.4	2.3
Chile	43.9	26.0	28.4	27.6	-40.8	-35.3	-37.2	-1.4
Grenada	37.4	71.8	76.9	76.5	91.8	105.5	104.3	2.3
Dominica	33.5	87.6	86.7	87.0	161.2	158.4	159.5	3.0
Jamaica	32.0	73.7	77.3	78.9	130.8	141.9	146.9	2.9
Belize	31.9	94.0	97.3	79.9	194.2	204.7	150.2	2.9
Uruguay	31.3	29.2	31.5	32.3	-6.5	0.9	3.3	0.1
Antigua and Barbuda	30.6	44.8	53.2	54.3	46.3	73.8	77.6	1.8
Costa Rica	30.0	27.4	26.3	29.2	-8.7	-12.3	-2.9	-0.1
Latin America and Caribbean	132.9	87.2	91.8	94.7	-34.4	-30.9	-28.7	-1.1
Regional gap [((Max/Min)-1)x100]	1,457.6	1,797.7	1,616.4	1,473.7	----	----	----	----

Elaborated by the authors using data from the Global Burden of Disease project (retrieved from: https://vizhub.healthdata.org/sdg/#0)

From 1990 to 2021, the number of HRH (physicians, nurses, and midwives) in LAC grew 145%, at a rate of 2.8% per year, from 1 physician and 2.1 nurses and midwives per 1 K inhab. in 1990 to 2.4 physicians and 4.8 nurses and midwives in 2021 (Table 2). In 1990, the countries with the highest density of physicians per 1 K inhab. were Cuba (3.3), Uruguay (1.8), Bahamas (1.8), Ecuador (1.6), and Barbados (1.6), whereas, in 2021, Cuba, Uruguay, Bahamas, Barbados, and Ecuador registered the highest density of physicians (10.3, 5.3, 5.1, 3.3 and 3.0 respectively). In contrast, the countries that in 1990 and 2021 registered less than one physician per 1 K inhab. were located mainly in the Caribbean. The regional gap ([((Max/Min)-1) x 100]) in the availability of physicians grew 33%, from 1,212.8% in 1990 to 1,614.2% in 2021. The highest density of nurses and midwives in 1990 was observed in the Bahamas (5.3), Cuba (5.1), Barbados (4.7), Suriname (4.3) and Antigua and Barbuda (3.6). In 2021, Cuba, Bahamas, Barbados, Antigua and Barbuda, and Brazil had the highest number of nurses and midwives (16.8, 15.7, 9.1, 7.6, and 6.7 respectively), while Nicaragua, Costa Rica, Haiti, Guatemala, and Argentina had the lowest density of nurses and midwives (0.7, 1.3, 1.6, 2.1 and 2.7 respectively). The regional gap in the availability of these resources grew 15%, from 1,912.1% in 1990 to 2,189.1% in 2021 (Table 2). The ratio of physicians to nurses and midwives remained unchanged in LAC (0.6), with important differences in Nicaragua (2.4) and Costa Rica (1.8) as the countries with the highest ratio and the countries with the lowest number of physicians per nurse and midwife (< 0.5). The regional gap in this indicator fell from 2,034.1% in 1990 to 1,776.9% in 2021 (Table 2).

**Table 2:** HRH endowment and evolution in LAC, 1990–2021

Country	Physicians per 1 K inhab. (A)				Nurses and midwives per 1 K inhab. (B)				Physicians-nurses and midwives ratio (C)				Relative change (1990–2021), %			Average annual growth rate (1990–2021), %		
	1990	2015	2019	2021	1990	2015	2019	2021	1990	2015	2019	2021	(A)	(B)	(C)	(A)	(B)	(C)
Haiti	0.3	0.5	0.6	0.6	0.9	1.4	1.5	1.6	0.3	0.4	0.4	0.4	135.3	74.5	34.8	2.7	1.8	0.9
Bolivia	0.6	1.9	2.2	2.3	1.2	3.8	4.3	4.7	0.5	0.5	0.5	0.5	274.0	285.4	-2.9	4.2	4.3	-0.1
Guatemala	0.5	1.3	1.4	1.6	0.7	1.8	1.9	2.1	0.7	0.7	0.8	0.8	221.4	194.0	9.3	3.7	3.4	0.3
Honduras	0.4	0.8	0.9	1.0	0.8	2.0	2.3	2.5	0.5	0.4	0.4	0.4	152.9	228.1	-22.9	2.9	3.8	-0.8
Peru	1.4	2.5	2.7	2.8	1.6	2.8	3.0	3.2	0.9	0.9	0.9	0.9	97.4	101.5	-2.0	2.1	2.2	-0.1
El Salvador	0.8	1.7	1.9	2.1	0.9	2.3	2.6	2.8	0.8	0.8	0.7	0.7	170.3	208.7	-12.4	3.2	3.6	-0.4
Brazil	0.7	1.7	1.9	2.0	2.5	5.6	6.3	6.7	0.3	0.3	0.3	0.3	178.5	163.0	5.9	3.3	3.1	0.2
Ecuador	1.6	2.6	2.9	3.0	1.1	2.5	2.7	2.9	1.5	1.1	1.1	1.0	91.4	177.2	-31.0	2.0	3.2	-1.2
Paraguay	0.6	1.1	1.3	1.4	1.3	2.3	2.6	2.7	0.5	0.5	0.5	0.5	113.5	112.0	0.7	2.4	2.4	0.0
Suriname	0.4	0.8	0.9	0.9	4.3	5.7	5.7	5.8	0.1	0.1	0.2	0.2	108.8	34.3	55.5	2.3	0.9	1.4
Colombia	1.1	2.0	2.2	2.4	1.6	2.8	3.1	3.3	0.7	0.7	0.7	0.7	110.6	107.7	1.4	2.4	2.3	0.0
Dominican Republic	0.7	1.4	1.5	1.5	2.6	4.2	4.5	4.7	0.3	0.3	0.3	0.3	107.4	80.0	15.2	2.3	1.9	0.4
Guyana	0.5	1.2	1.3	1.4	1.5	2.9	3.2	3.4	0.4	0.4	0.4	0.4	155.6	126.5	12.8	3.0	2.6	0.4
Nicaragua	0.6	1.4	1.6	1.8	0.3	0.6	0.7	0.7	2.2	2.4	2.4	2.4	207.4	178.0	10.6	3.6	3.2	0.3
Mexico	1.1	2.4	2.5	2.7	2.1	5.2	5.6	5.9	0.5	0.5	0.5	0.4	133.8	178.6	-16.1	2.7	3.3	-0.5
Venezuela	0.9	1.3	1.2	1.3	2.3	3.8	3.7	3.8	0.4	0.3	0.3	0.3	34.0	65.3	-19.0	0.9	1.6	-0.7
Panama	1.1	2.2	2.4	2.6	2.3	4.3	4.8	5.1	0.5	0.5	0.5	0.5	125.6	119.7	2.7	2.6	2.5	0.1
Argentina	1.5	2.4	2.6	2.7	1.6	2.4	2.6	2.7	0.9	1.0	1.0	1.0	86.9	69.2	10.5	2.0	1.7	0.3
Trinidad and Tobago	0.3	0.7	0.7	0.7	2.4	5.1	5.4	5.7	0.1	0.1	0.1	0.1	116.2	139.8	-9.9	2.4	2.8	-0.3
Cuba	3.3	8.6	9.6	10.3	5.1	14.0	15.6	16.8	0.7	0.6	0.6	0.6	207.2	232.5	-7.6	3.6	3.8	-0.2
Bahamas	1.8	4.5	4.8	5.1	5.3	13.8	14.9	15.7	0.3	0.3	0.3	0.3	176.0	195.5	-6.6	3.2	3.4	-0.2
Saint Vincent and the Grenadines	0.5	1.4	1.6	1.7	1.9	3.9	4.3	4.5	0.3	0.4	0.4	0.4	207.1	143.6	26.1	3.6	2.8	0.7
Barbados	1.6	3.0	3.2	3.3	4.7	8.3	8.8	9.1	0.3	0.4	0.4	0.4	109.6	93.6	8.3	2.3	2.1	0.2
Saint Lucia	0.7	1.8	2.1	2.2	2.3	5.2	5.8	6.1	0.3	0.4	0.4	0.4	231.0	167.3	23.8	3.8	3.1	0.7
Chile	0.8	1.3	1.4	1.4	1.4	4.9	5.7	6.3	0.6	0.3	0.2	0.2	66.2	352.0	-63.2	1.6	4.8	-3.1
Grenada	0.5	1.4	1.6	1.7	2.1	4.0	4.3	4.5	0.3	0.4	0.4	0.4	215.4	116.3	45.8	3.7	2.4	1.2
Dominica	0.7	1.6	1.8	1.9	2.0	4.1	4.5	4.8	0.3	0.4	0.4	0.4	173.4	136.0	15.9	3.2	2.7	0.5
Jamaica	0.9	1.9	2.1	2.2	2.6	4.7	5.1	5.3	0.4	0.4	0.4	0.4	136.5	103.2	16.4	2.7	2.2	0.5
Belize	0.4	1.2	1.3	1.5	1.3	3.3	3.8	4.0	0.3	0.4	0.4	0.4	294.3	210.3	27.1	4.4	3.6	0.8
Uruguay	1.8	4.3	4.9	5.3	2.3	5.3	6.0	6.4	0.8	0.8	0.8	0.8	197.3	181.2	5.7	3.5	3.3	0.2
Antigua and Barbuda	1.2	2.3	2.5	2.6	3.6	6.6	7.2	7.6	0.3	0.4	0.3	0.3	115.9	109.9	2.9	2.4	2.3	0.1
Costa Rica	1.4	2.1	2.2	2.3	0.7	1.1	1.2	1.3	2.2	1.9	1.9	1.8	59.2	91.2	-16.7	1.5	2.0	-0.6
Latin America and Caribbean	1.0	2.0	2.2	2.4	2.1	4.4	4.8	5.1	0.6	0.6	0.6	0.6	144.5	142.5	-2.6	2.8	2.8	-0.1
Regional gap [((Max/Min)-1)x100]	1,212.9	1,604.5	1,612.3	1,614.2	1,912.1	2,244.4	2,201.9	2,189.1	2,034.1	1,713.1	1,763.4	1,776.9						

Elaborated by the authors using data from the Global Burden of Disease project (retrieved from: http://ghdx.healthdata.org/record/ihme-data/gbd-2017-health-related-sdgs-1990-2030)

**Fig. 1:**
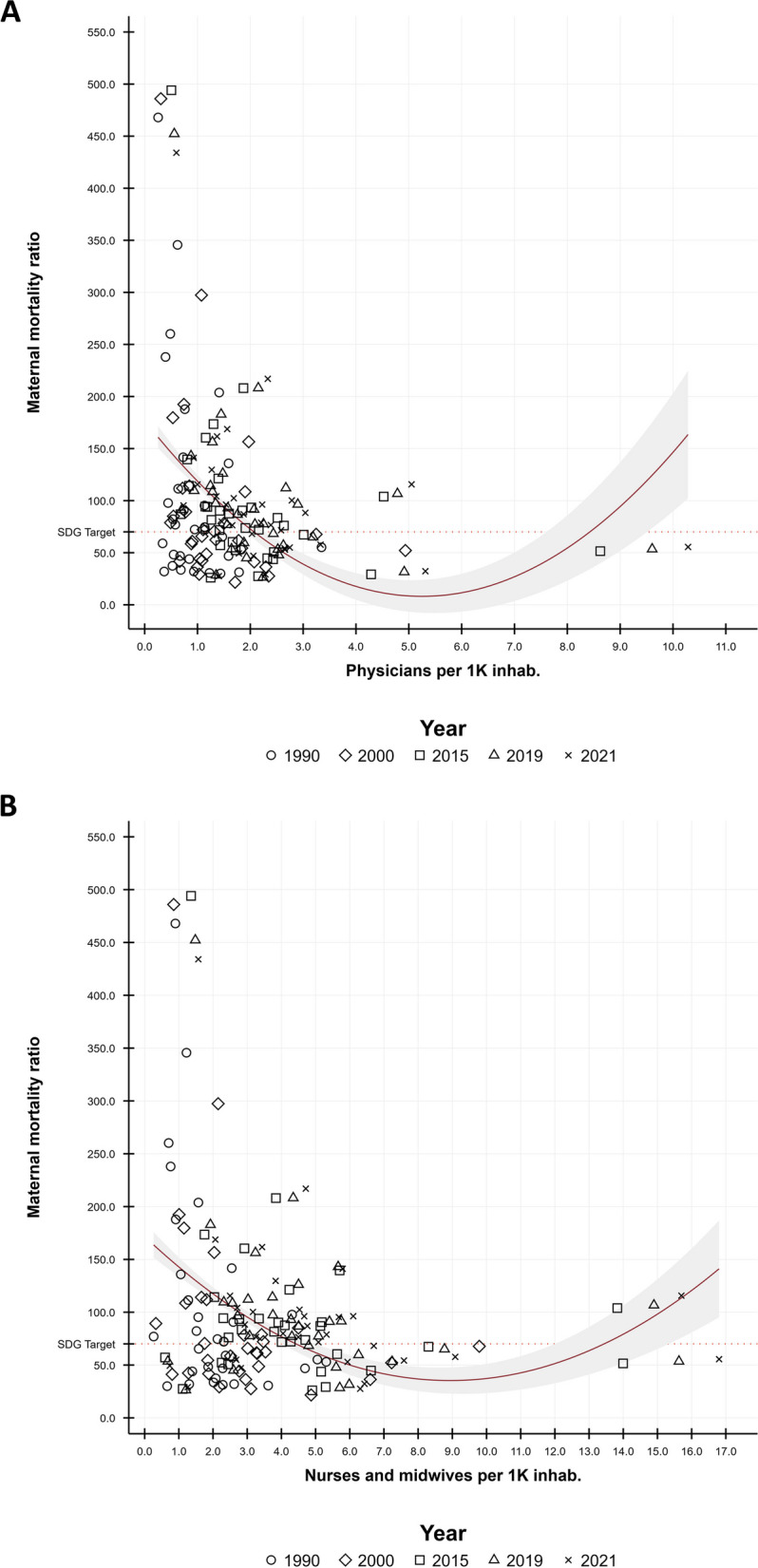
Levels and changes in maternal mortality ratio and availability of HRH in LAC, 1990–2021. **A** Physicians per 1 K inhab. **B** Nurses and midwives per 1 K inhab.  Note: Elaborated by the authors using data from the Global Burden of Disease project (retrieved from: https://vizhub.healthdata.org/sdg/#0 and http://ghdx.healthdata.org/record/ihme-data/gbd-2017-health-related-sdgs-1990-2030). In red solid line we present the quadratic prediction (with CI95%) estimated by the regression between MMR and HRH availability

The relationship between MMR and the number and evolution during the 32 years analyzed of the availability of HRH in LAC was nonlinear and heterogeneous (Fig. 1, panels A and B): (i) The lowest levels of MMR (< 70 -meta 3 of the SGDs-) were concentrated in countries with numbers of between one and two physicians and one to six nurses and midwives per 1 K population. (ii) At these HRH availabilities, some countries recorded 20 times higher levels of MMR, while other countries with MMR levels < 70 recorded physician and nurse-midwife availability of up to 11 and 17 times higher, respectively. (iii) Despite most Latin countries recording increases in HRH availability and reductions in MMR, those countries in the Caribbean experienced the opposite (increases in HRH and MMR). Reductions > 25% in MMR were observed with increases of 60–275% in physician density (Chile and Bolivia, respectively) and 100–275% in nurse and midwife density (Peru and Bolivia, respectively).

**Fig. 2:**
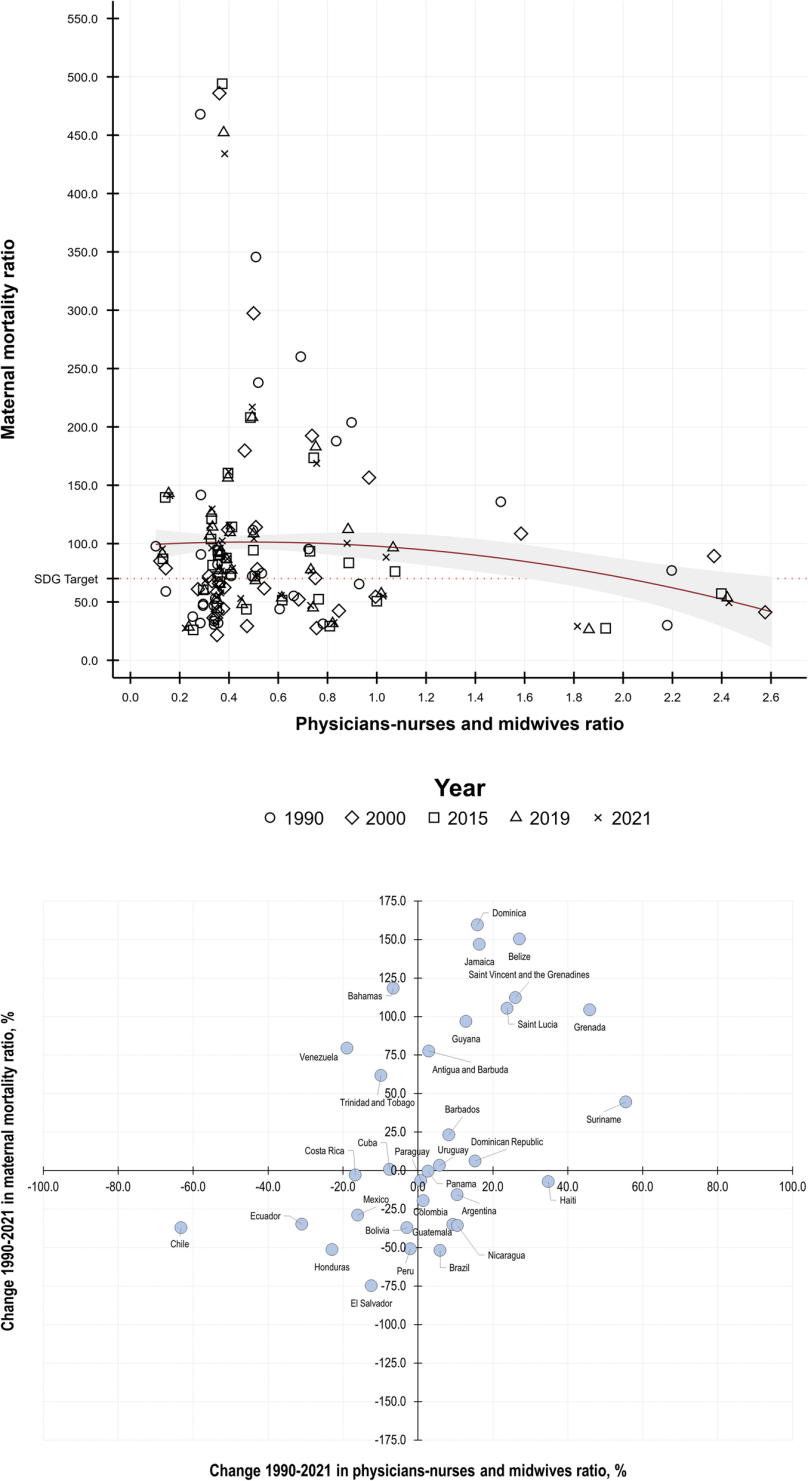
Levels and changes in maternal mortality ratio and physician-to-nurse and midwife ratios in LAC, 1990–2021. **A** Relationship between levels 1990–2021. **B **Relationship between changes 1990–2021. Note: Elaborated by the authors using data from the Global Burden of Disease project (retrieved from: https://vizhub.healthdata.org/sdg/#0and http://ghdx.healthdata.org/record/ihme-data/gbd-2017-health-related-sdgs-1990-2030). In red solid line we present the quadratic prediction (with CI95%) estimated by the regression between MMR and HRH availability

Along the same lines, the number of physicians per nurse and midwives also had a nonlinear and heterogeneous relationship with MMR (Fig. 2). Although during 1990–2021, the levels of MMR < 70 (target 3 of the SGDs) were concentrated in values of the ratio of physicians per nurse and midwife of 0.2 to 0.8, we observed countries that, in that range, recorded levels of MMR > 450; as well as countries that, with 2.6 physicians per nurse and midwife, recorded levels of MMR < 70 (Fig. 2, Panel A). When correlating the change in MMR and that ratio, five particular patterns stood out (Fig. 2, Panel B): (i) Again, in most of the Caribbean countries, there was an increase in MMR, regardless of the growth or reduction in the number of physicians per nurse and midwife; (ii) in Costa Rica, Cuba, Paraguay, Panama and Uruguay, MMR did not vary in the face of changes in the physician-nurse and midwife ratio; (iii) in Colombia, Bolivia and Peru MMR decreased even though the referred ratio was practically unchanged; iv) Mexico and El Salvador recorded a 15% reduction in the number of physicians per nurses and midwives; however, the reduction in MMR was notably different (25% and 75% respectively); v) Brazil and Honduras reduced by 50% and while Nicaragua, Ecuador and Chile reduced their MMRs by around 35%, their changes in the above ratio were notably different (6%, -23%, 11%, -31% and 63% respectively).

## Discussion

Interest in the analysis of HRH and its relationship with the field of sexual and reproductive health, including maternal death in LAC, is still scarce. The classic studies by Annand and Barnighausen (2004) that related these two aspects to other regions of the [26] were not replicated in LAC. This omission is even more remarkable if we consider that there are now open-access information bases that would allow this type of analysis. This paper offers a first example of how these analyses could be performed and suggests that high staffing densities could be related to more significant reductions in maternal mortality in the region. However, it is necessary to consider the role of other resources that health systems contain that could positively reduce maternal mortality.

To function, health systems require a variety of resources, including human, financial, technological, drugs and infrastructure. HRH enables systems to achieve population care goals. The post-2015 agenda for sustainable development calls for a drastic reduction of the maternal mortality [11] and recognizes the strategic role of HRH in organizing and managing all other resources and achieving system goals such as coverage, equity, efficiency [27], and quality of care [28].

The data presented shows the enormous heterogeneity of the response of the regional health systems to the challenge posed by maternal mortality in the region. Although most countries articulated specific programs to achieve the reduction committed by all countries through the MDGs, not all had the same capacity to reduce it, and practically none met the target [29]. Initially, cases in the antipodes can be highlighted, such as Venezuela [30], which had the lowest increase in health personnel in the entire region and a significant increase in maternal mortality. On the other hand, Bolivia stood out with the most significant increase in health personnel and a substantial reduction in MMR. Furthermore, we found the entire block of English Caribbean countries with significant increases in the number of health personnel that do not explain the increases in the MMR during the period.

Following the systemic perspective, it is important to note the potential role played by the availability of other resources for health in the MMR levels. According to OECD data, by 2019 [31], among the countries with the lowest per capita expenditure on health, below 1,000 USD PPP annually, in the region were Haiti, Venezuela, Honduras, Belize, Nicaragua, Guatemala, Jamaica, St. Vincent & Grenada, Bolivia, Guyana, Dominica, St. Lucia, Peru, and El Salvador. According to the data analyzed, among the countries that achieved the greatest MMR reductions, we found Bolivia, El Salvador, Honduras, and Peru, which suggests that their low relative investment could have been used efficiently to reduce MMR. On the other hand, the English Caribbean countries Bahamas, Trinidad & Tobago, Suriname, Saint Kitt and Nevis, Barbados, and Antigua & Barbuda, which had per capita expenditure above 1,000 USD PPP, did not achieve significant reductions in MMR.

Another relevant resource is the availability of hospital beds. OECD data concerning this indicator show that Caribbean countries such as Barbados, Antigua & Barbuda, Grenada, Suriname, Trinidad & Tobago, and the Bahamas, had above 3 beds per 1,000 inhab. in 2014, while continental countries in the region, such as Colombia, Peru, Ecuador, El Salvador, Bolivia, and Mexico showed a ratio of less than 2 beds per 1 K inhab. Even Costa Rica was in this group of countries. There is likely no relationship between the availability of hospital beds and MMR if delivery care in these countries does not preferably take place in hospitals, although it is important to consider that countries such as Mexico, Peru, and Colombia do have a preference for hospital care in childbirth [32].

A third, more specific indicator is that care delivery in most countries occurs in health institutions rather than at home or in other settings. The region shows an average of 90% of delivery care performed in health institutions with some variations. Caribbean countries such as Guyana, Suriname and Belize show the lowest proportions (between 92 and 95%) of delivery care in health institutions while Cuba, Argentina, Colombia, and Costa Rica reached levels close to 100%.

As in HRH’s case, there is no direct relationship between the exemplified resources and MMR. However, it is important to note that only some countries (particularly Costa Rica) have managed to reduce MMR to a minimum without increasing the number of available resources. The answer can be found in the primary healthcare model historically prevalent in that country [33]. Other countries such as Honduras, El Salvador and Peru, whose health systems are not based on a primary health care model, depend on short-term programs to achieve temporary success [34]. In the case of the Caribbean countries we have already highlighted, the growth of maternal mortality may be related to the fall in financial and human resources. Moreover, according to UNFPA, in this region the increase in obesity, diabetes, high prevalence of HIV and adolescent pregnancy are primary determinants of high maternal mortality [35]. As the WHO points out in its 2016 report, the issue with health resources, including HRH, does not imply that we will have better health outcomes from their greater availability since various aspects play a role, including the type of training, the type of functions they perform, the level of care at which they operate, productivity and quality in the execution of tasks [11].

Focusing back on HRH, the data on the relationship between HRH availability and MMR is an example of what we might be projecting regarding health problems using encompassing views. It is important to identify the areas in which HRH planning, training, and distribution/availability decisions impact on a substantive level and the approaches that underlie their training and performance. Beyond the relationships between variables, we also need to consider the characteristics of the model of obstetric care. The current model, which is widely prevalent in LAC, intervenes massively in women’s bodies, creating a series of risks for both mothers and children [36]. For this reason, in recent years the WHO has recommended promoting low-intervention models of obstetric care, with the participation of a diversity of qualified service providers, which focus on the natural process and the emotional needs of women [37]. The change of model could help to reduce maternal mortality in all settings, particularly in those with scarce financial resources and low availability of human resources. Thus, we believe it may be possible to model, deconstruct, and reconstruct obstetric care to incorporate perspectives of rights, cultural inclusion, and care for the environment, among others [38–40]. The fragmentation of the approach to health and the excessive emphasis on disease by the HRH impacts the health system, the educational system, and the labor market. Finally, the area of construction of process and outcome indicators is identified, which, in line with the above, are modeled by the way health is conceptualized and the way health providers put in practice their skills. The previous areas, as effects of the process of planning, training, and distribution/availability of HRH, have a direct relationship with how equity and quality of health care are constructed from the conceptual, but above all from the operational level [41].

The results presented in this commentary should be interpreted while considering several limitations, mainly related to using the GBD data, as previously documented field [41–44], and the ecological descriptive analysis performed. First, there is an ecological fallacy in interpreting results due to the use of aggregate data from the GBD study, which are subject to statistical modeling and do not reflect individual-level data. In this sense, the relationships evidenced only suggest the existence of statistical associations and not causal relationships. Second, the GBD estimates depend on the quality of the data provided by the countries, which could be of great concern in countries with limited availability of data of acceptable quality. Third, although conceptual arguments justify the existence of the relationships explored [26], it is important to recognize the omission of relevant variables in the analysis performed, such as, for example, the level of investment of specific resources for maternal health care.

## Conclusions

Since the signing of the MDGs, LAC countries have embarked on strategies to reduce MMR. However, despite the effort and resources allocated, the achievements of these strategies have generated fewer than expected dividends in most countries. HRH were considered essential to push the strategies, but their relationship with the achievements of maternal mortality reduction needs to show in the data analyzed a clear relationship. This should lead us to reflect on the role of HRH in a health system and what relationship they should have with other resources to enhance their effect. Undoubtedly, the strategies in each country should be reviewed to strengthen the levels of training, the conceptualization of maternal health care and childbirth, the geographic allocation, and the type of units where this care should be offered. It is important to ensure that obstetric care maintains the highest standards of quality and protects the rights of women and their children.

## Data Availability

The final database underlying this study has been uploaded to Figshare and is freely accessible using the following link: 10.6084/m9.figshare.24636723.

## Abbreviations

MMR: Maternal Mortality Ratio
MDGs: Millennium Development Goals
HRH: Human Resources for Health
SDGs: Sustainable Development Goals
UHC: Universal Health Coverage
LAC: Latin American and Caribbean Region
GBD: Global Burden of Disease
